# The role of industrial actors in the circular economy for critical raw materials: a framework with case studies across a range of industries

**DOI:** 10.1007/s13563-022-00304-8

**Published:** 2022-02-21

**Authors:** Alexander Cimprich, Steven B. Young, Dieuwertje Schrijvers, Anthony Y. Ku, Christian Hagelüken, Patrice Christmann, Roderick Eggert, Komal Habib, Atsufumi Hirohata, Alan J. Hurd, Min-Ha Lee, David Peck, Evi Petavratzi, Luis A. Tercero Espinoza, Patrick Wäger, Alessandra Hool

**Affiliations:** 1 School of Environment, Enterprise and Development (SEED), University of Waterloo, 200 University Ave West, Waterloo, Ontario N2L3G1 Canada; 2 WeLOOP, 254 rue du Bourg, 59130 Lambersart, France; 3 NICE America Research, Mountain View, CA 94043 USA; 4 National Institute of Clean-and-Low-Carbon Energy, Beijing, 102209 People’s Republic of China; 5 Umicore AG & Co. KG, Rodenbacher Chaussee 4, 63457 Hanau, Germany; 6 KRYSMINE, 1163, rue de Savigny, 45640 Sandillon, France; 7 Department of Economics & Business, Colorado School of Mines, Golden, CO 80401 USA; 8 Department of Electronic Engineering, University of York, Heslington, York, YO10 5DD UK; 9 Los Alamos National Laboratory, PO Box 1663, Los Alamos, NM 87545 USA; 10 Korea Institute of Industrial Technology, (KITECH), KITECH North America, 2833 Junction Ave. Suite 207, San Jose, CA 95134 USA; 11 Faculty of Architecture and the Built Environment, Delft University of TechnologyArchitectural Engineering and TechnologyDelft University of Technology (TU Delft), Building 8Julianalaan 134, 2628BL Delft, The Netherlands; 12 Environmental Science Center, Decarbonisation and Resource Managemental, British Geological Survey, Nottinghamshire NG12 5GG Keyworth, UK; 13 Competence Center Sustainability and Infrastructure Systems, Fraunhofer Institute for Systems and Innovation Research ISI, Breslauer Straße 48, 76139 Karlsruhe, Germany; 14 ESM Foundation, Junkerngasse 56, 3011 Bern, Switzerland; 15 Swiss Federal Laboratories for Materials Science and Technology, Technology & Society Laboratory, Lerchenfeldstrasse 5, CH-9014 St. Gallen, Switzerland

**Keywords:** Critical raw materials, Material criticality, Supply security, Circular business models, Circularity strategies, Industrial actors

## Abstract

In this article, we explore concrete examples of circularity strategies for critical raw materials (CRMs) in commercial settings. We propose a company-level framework for systematically evaluating circularity strategies (e.g., material recycling, product reuse, and product or component lifetime extension) in specific applications of CRMs from the perspectives of specific industrial actors. This framework is applied in qualitative analyses—informed by relevant literature and expert consultation—of five case studies across a range of industries: (1) rhenium in high-pressure turbine components, (2) platinum group metals in industrial catalysts for chemical processing and oil refining, (3) rare earth permanent magnets in computer hard disk drives, (4) various CRMs in consumer electronics, and (5) helium in magnetic resonance imaging (MRI) machines. Drawing from these case studies, three broader observations can be made about company circularity strategies for CRMs. Firstly, there are multiple, partly competing motivations that influence the adoption of circularity strategies, including cost savings, supply security, and external stakeholder pressure. Secondly, business models and value-chain structure play a major role in the implementation of circularity strategies; business-to-business models appear to be more conducive to circularity than business-to-consumer models. Finally, it is important to distinguish between closed-loop circularity, in which material flows are contained within the “focal” actor’s system boundary, and open-loop circularity, in which material flows cross the system boundary, as the latter has limited potential for mitigating material criticality from the perspective of the focal actor.

**Supplementary Information:**

The online version contains supplementary material available at 10.1007/s13563-022-00304-8.

## Introduction

As part of the European Institute of Innovation and Technology (EIT) RawMaterials consortium, the International Round Table on Materials Criticality (IRTC) was established to convene international experts on the subject of material criticality. Critical raw materials (CRMs) have attracted growing research and policy interest given the diversity of these materials used in modern technologies and the complexity of globalized supply chains. Many CRMs play a key role in such essential applications as information technology, low-carbon energy systems, clean mobility, and healthcare (see, e.g*.*, Achzet and Helbig (2013), Erdmann and Graedel (2011), European Commission. (2020,2017,2014,2010), Graedel et al. (2012), Graedel and Reck (2016), Helbig et al. (2016), National Research Council. (2008), and U.S. (2018).

As described in a recent review of over 40 criticality assessment methods (Schrijvers et al. 2020), material criticality can be broadly conceptualized as a combination of the probability and consequences of supply disruptions (resulting from a multitude of factors such as production concentration, trade barriers, geopolitical instability, and by-product dependency) of a given material, for a given stakeholder, within a given timeframe. The context- and scope-dependent nature of the concept is reflected in the wide variety of criticality assessment methods available in the literature, resulting in diverging conclusions of which materials are (most) “critical” (Schrijvers et al. 2020).

One set of strategies for responding to the problem of material criticality is aimed at reducing primary material demand and maximizing overall resource efficiency using the “circular economy” (CE) concept (Gaustad et al. 2018). As with the criticality concept, there are a wide variety of perspectives on the CE concept (Ghisellini et al. 2016; Blomsma and Brennan 2017; Kirchherr et al. 2017; UNEP International Resource Panel 2018), with circularity strategies like “recycling,” “reuse,” and “remanufacturing” often lacking a clear and consistent terminology (Blomsma and Tennant 2020). In an effort to systematize the CE discourse, Blomsma and Tennant (2020) developed the Resource States framework, which is grounded in aspects of life cycle thinking. The notion of a product life cycle—from primary material extraction through product manufacturing, product use, and product end-of-life—is one of the basic conceptual underpinnings of life cycle assessment (LCA), a widely used and internationally standardized methodological framework for evaluating the resource use and environmental impacts associated with a given product or service (ISO 2006a, b). Through this lens, Blomsma and Tennant (2020) distinguish three “resource states” for which different circularity strategies can be implemented: *particles* (i.e., elements, substances, molecules, and materials), *parts* (i.e., components, modules, and subassemblies), and *products* (i.e., finished goods). In the work presented here, we adapt this framework to examine circularity strategies for CRMs.

The CE discourse to date has focused primarily on bulk materials used in large quantities throughout the economy (e.g., cement, copper, iron, aluminum, plastics, and paper) (Gaustad et al. 2018; Tercero Espinoza et al. 2020). Despite having unique and desirable properties that often make them challenging to substitute (Graedel et al. 2015), CRMs are often used in comparatively small amounts and consequently tend to be overlooked from the CE perspective, especially when using mass-based indicators (e.g., recycling targets) (Talens Peiró et al. 2011; UNEP International Resource Panel 2011; Nassar et al. 2015)—although some CE models, such as the EU Material System Analysis, examine individual material flows (Tercero Espinoza et al. 2020). As the production of each mineral or metal has its specificities in terms of the nature of the ore extracted, of the processes used to obtain marketable products, of related inputs and outputs (emissions, waste), of the markets addressed, and of their dynamics, it is important to develop mineral- or metal-specific knowledge. Although much data and knowledge exists with respect to main metals, data and knowledge on CRMs is very sparse. To the extent that CE approaches for CRMs have been considered, these considerations have been largely limited to material recycling—neglecting other circularity strategies such as product reuse and lifetime extension through various forms of product repair, refurbishment, and remanufacturing (Gaustad et al. 2018; Bobba et al. 2020; Tercero Espinoza et al. 2020). In their review, Gaustad et al. (2018) also note that “[t]he high-level perspective taken by most [material criticality] assessments (global or national) makes it difficult and potentially inappropriate for firms to directly apply the findings to inform their supply chain management strategies” (p. 25). However, individual companies may profit from the adoption of circularity strategies focusing on CRMs, e.g*.*, to address security of supply concerns, to control or reduce costs, or for reputational reasons. Therefore, the focus of this article is on the examination of actual examples of company-level circularity strategies for CRMs.

This article contributes to the literature in two key ways: Firstly, a systematic review of company-level CE approaches (Roos Lindgreen et al. 2020) indicates that this relatively new area of research (with only 6 publications prior to 2016) could benefit from further consideration of real-life factors (e.g*.*, company goals, decision-making contexts, and barriers) for the implementation of circularity strategies. These real-life factors are embedded in our framework and made explicit in our analysis. Secondly, we extend the analysis of circularity strategies to CRMs, thus going beyond common mass-based indicators. Practical industrial experience is combined with a company-level CE framework^1^ for systematically evaluating company circularity strategies for CRMs. This company-level approach recognizes the nuances of both the material criticality and CE concepts, while supporting concrete steps towards managing material criticality in business practice.

To date, public descriptions of company circularity strategies have been primarily qualitative and anecdotal in nature. This is not because programs by companies lack definition or rigor; rather, the dearth of public data on mass flows, economics, and business models is due to the commercial implications of sharing this information. Regardless of whether a given circularity effort was successful or not, detailed information on company operations is generally considered confidential and proprietary. Successful examples are sometimes publicized on a case-by-case basis, and even in those cases, details can be sparse. In this article, we review five case studies of successful circularity efforts that have been described in the literature and introduce some details that have not previously been elucidated. The core contribution of this article involves clarifying the underlying circularity strategies that underpin the business cases in these examples.

The article is structured as follows. In the “Materials and methods” section, we describe the company-level CE framework—an adaptation of the Resource States framework developed by Blomsma and Tennant (2020)—that we use to examine circularity strategies in specific applications of CRMs from the perspectives of specific “industrial actors.” We then briefly outline five case studies—in which we apply this framework—across a range of industries where circularity strategies have been implemented for CRMs in commercial practice. These cases are as follows: (1) rhenium in high-pressure turbine components, (2) platinum group metals in industrial catalysts for chemical processing and oil refining, (3) rare earth permanent magnets in computer hard disk drives, (4) various CRMs in consumer electronics, and (5) helium in magnetic resonance imaging (MRI) machines used for medical imaging. Qualitative analyses of these case studies—informed by relevant literature and expert consultation—are presented in the “Analysis of case studies” section. In these analyses, we map the implemented circularity strategies onto our framework, while discussing the motivations, enabling (or inhibiting) factors, and outcomes of the circularity strategies (with respect to material criticality) from the perspective of the “focal” industrial actor. In the “Discussion” section, we highlight broader observations across the case studies, and finally, we close in the “Conclusions and outlook” section with an outlook on areas for future research.

## Materials and methods

We developed our framework and case studies through an iterative process beginning at the EU Raw Materials Week in Brussels in November 2019. Further explanation of this process is provided in an Online Resource.

### Company-level CE framework

**Fig. 1:**
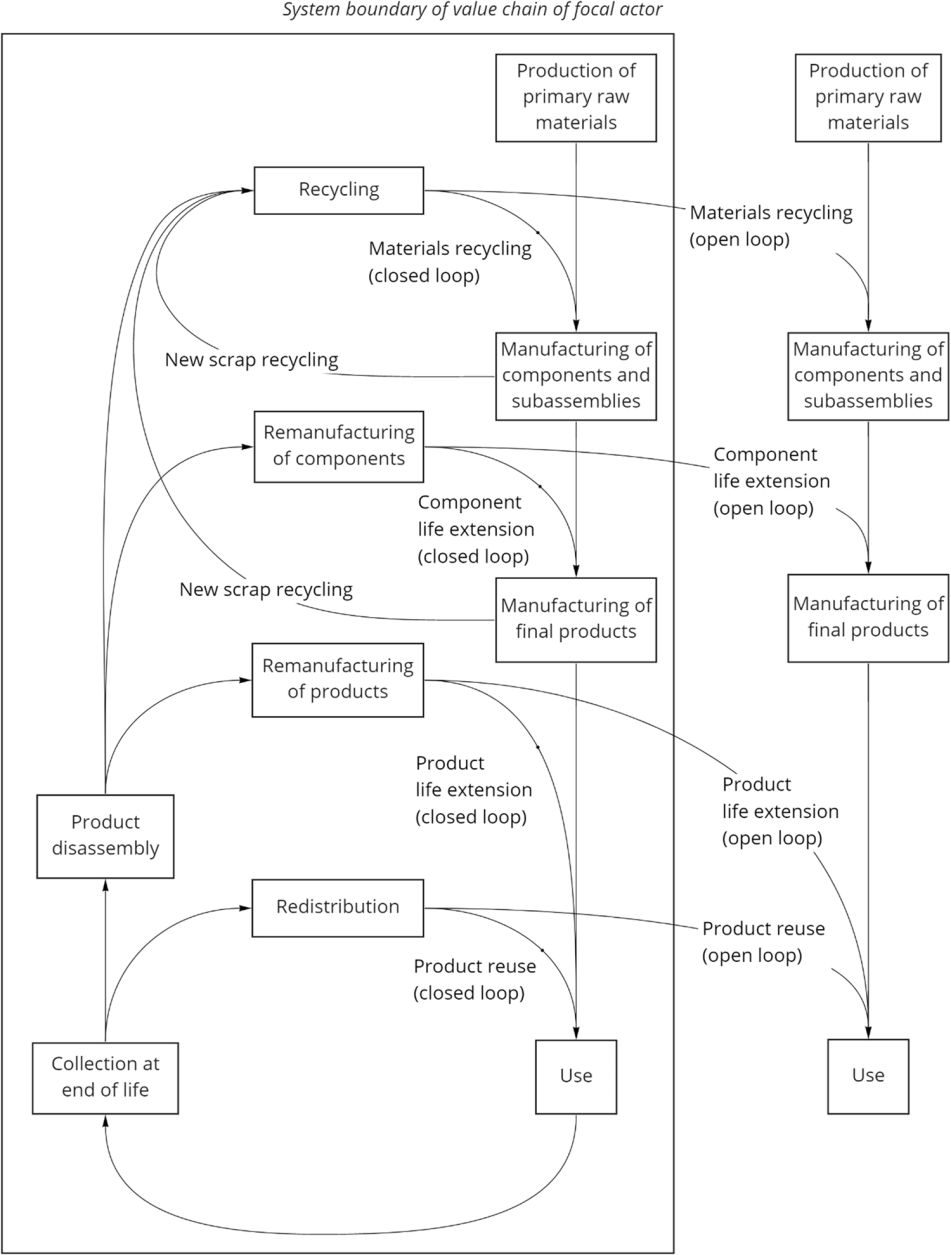
Company-level framework for evaluating circularity strategies for critical raw materials ( adapted from Blomsma and Tennant (2020))

The general framework used herein is an adaptation of the Resource States framework developed by Blomsma and Tennant (2020), which is structured around the value chain of a specific application of a CRM (or group of CRMs) from the perspective of a specific industrial actor—the “focal” actor (Fig. 1). We use this framework for several reasons. First, the development of the Resource States framework was informed by a widely cited narrative review (with 343 citations as of December 8, 2021, according to the Scopus database) previously conducted with the same lead author (Blomsma and Brennan 2017). Further to this, the Resource States framework is strengthened by its grounding in *life cycle thinking*. The notion of a product “life cycle”—from primary material extraction through product manufacturing, product use, and product end-of-life—is one of the basic conceptual underpinnings of life cycle assessment (LCA), a widely used and *internationally standardized* methodological framework, tested, and demonstrated through decades of practical application, for evaluating the resource use and environmental impacts associated with a given product or service (ISO 2006a, b). Although LCA is not our focus in this article, life cycle thinking nonetheless provides a well-developed conceptual foundation to build upon. Through this lens, Blomsma and Tennant (2020) consider a comprehensive set of circularity strategies^2^ that can be implemented for three different “resource states”: *particles* (i.e., elements, substances, molecules, and materials), *parts* (i.e., components, modules, and subassemblies), and *products* (i.e., finished goods). Finally, the Resource States framework, despite encompassing a comprehensive set of circularity strategies, is remarkably clearly arranged and compact—with both the original version (Fig. 1 in Blomsma and Tennant (2020)) and our adaptation (Fig. 1 in this article) fitting neatly on a single page.

As illustrated in Fig. 1, we adapt the Resource States framework to systematically evaluate circularity strategies in a specific application of a CRM (or group of CRMs) from the perspective of a specific “industrial actor.” We use the term “actor” in the broadest sense of “one that takes part in any affair”, and the term “*industrial* actor” to refer to any company, organization, or institution in any industry or sector. The specific industrial actor in question is referred to as the “focal” actor (Fig. 1). The notion of the “focal” actor in our framework recognizes the fundamental context-dependency of the material criticality concept as highlighted in the previously cited reviews by Gaustad et al. (2018) and Schrijvers et al. (2020). Accordingly, our adaptation of the Resource States framework is structured around the value chain of the CRM(s) in question. Each labeled box represents a value chain stage with corresponding processes and output flows. The first three stages are *production of primary raw materials*, *manufacturing of components and subassemblies*, and *manufacturing of final products*—corresponding to the resource states of “particles,” “parts,” and “products,” respectively, in the originally published version of the Resource States framework (Blomsma and Tennant 2020).

Using this terminology, we distinguish circularity strategies based on the flows and corresponding processes avoided by these strategies. Product redistribution and reuse—which Blomsma and Tennant (2020) note mean “direct” or “as-is” product reuse without any form of repair, refurbishment, or remanufacturing—avoids *manufacturing of final products* (along with all upstream value-chain stages). Product lifetime extension involving some form of product repair, refurbishment, or remanufacturing avoids *manufacturing of final products*. Component lifetime extension through remanufacturing processes avoids *manufacturing of components and subassemblies*. Finally, material recycling—from “new scrap” or from disassembled end-of-life products—avoids *production of primary raw materials*.

As recognized by Blomsma and Tennant (2020), circularity strategies can form “open loops” or “closed loops.” As outlined in Table 1, these terms can be defined in different ways—e.g*.*, from a supply chain management perspective or from an “industrial ecology” perspective. Given that our adaptation of the Resource States framework takes the perspective of a focal industrial actor, we use the company-oriented conceptualization of open and closed loops from the field of supply chain management (as discussed by Kalverkamp and Young (2019)). Accordingly, material flows crossing the focal actor’s system boundary and entering another industrial actor’s value chain are termed open loop, whereas material flows contained within the focal actor’s system boundary are termed closed loop.

**Table 1:** Definitions of closed-loop and open-loop circularity in different contexts and in relation to different system boundaries

System boundary definition	Closed-loop circularity	Open-loop circularity
System boundaries defined by the focal actor’s value chain (as applied in Supply Chain Management (Kalverkamp and Young (2019)) and this article)	The secondary material is used in similar or different applications, produced by the same industrial actor as the user of the primary material	The secondary material is used in similar or different applications, produced by a different industrial actor as the user of the primary material
System boundaries defined by material functionality (as applied in the field of industrial ecology, especially with a focus on metals recycling (Dubreuil et al. 2010; Graedel et al. 2011))	The material is recycled back into similar or different applications, while maintaining its primary functionality	The material ends up as a “tramp” element in other recycled materials, while losing its functionality
System boundaries defined by application (as applied in the field of industrial ecology, considering a wide range of materials, e.g*.*, Schrijvers et al. 2016)	The material is recycled back into similar applications, where it provides the same functionality (e.g*.*, the use of recycled plastic in bottles)	The material is recycled back into different applications, where it provides a different functionality (e.g*.*, recycled plastics used in aggregates)

### Selection of case studies

The framework illustrated in Fig. 1 is applied in qualitative analyses of the five case studies outlined in Table 2. As elaborated in the Online Resource, these case studies were ultimately selected based on two key criteria: (1) coverage of a range of industries, CRMs, and circularity strategies and (2) sufficient information—from academic literature and/or company and government reports, along with personal communication with industry experts—to support our analyses. With a focus on the company level, information was obtained from company reports and expert informants, both inside companies and externally. Questions focused on company supply-chain structure, concerns around CRMs, and control of CRM processes. Further details are provided in the Online Resource. In each case study, we map the implemented circularity strategies onto our framework, indicate how supply chain stages are controlled by the focal actor, and discuss the motivations, enabling (or inhibiting) factors, and outcomes of the circularity strategies (with respect to material criticality) from the perspective of the focal actor.

**Table 2:** Outline of cases

Value chain for…	Main CRM(s) of concern*	Focal industrial actor	Data sources
Superalloys used for turbine blades and discs in the high-pressure, high-temperature section of jet engines and gas turbines	Rhenium	Jet engine manufacturer (e.g*.*, Cannon Muskegon, General Electric, Safran (and their Joint-Venture CFM International, Rolls Royce, or Pratt & Whitney)	Relevant literature (Lee et al. 2008; Fink et al. 2010; Dasan et al. 2011; Konitzer et al. 2012; Ku and Hung 2014; Srivastava et al. 2014, 2016; Rodrigues Vieira and Lavorato Loures 2016; Schulz et al. 2017; Rezaei Somarin et al. 2018; USGS 2019, 2020, 2021; Kesieme et al. 2019)
Chemical processing catalysts	Platinum group metals (PGMs)—principally platinum, palladium, and rhodium	Catalyst user (chemical plant or oil refinery)	Relevant literature (Hagelüken 2008, 2012, 2018, 2019, 2020; Hagelüken and Meskers 2010; Hagelüken et al. 2016; Mudd et al. 2018; Rasmussen et al. 2019; European Commission 2020; Johnson Matthey 2020; Yuan et al. 2020)
Permanent magnets in computer hard disk drives	Rare earth elements (neodymium and dysprosium)	Magnet manufacturer (Hitachi Group)	Relevant literature (Hagelüken and Meskers 2010; Binnemans et al. 2013; Sprecher et al. 2014; Habib et al. 2015; Constantinides 2016; Yang et al. 2017; Lixandru et al. 2017) Publicly available information from the company (Baba et al. 2013; Nemoto et al. 2019; Harada and Nemoto 2020) Personal communication with company representatives (Dr. Yasushi Harara and Dr. Takeshi Nemoto)
Consumer electronics	CRMs** prioritized based on Material Impact Profiles (Apple Inc. 2019a)	Consumer electronics manufacturer (Apple Inc.)	Relevant literature (Nokia Corp. 2008; Hagelüken and Meskers 2010; Tanskanen 2013; Cucchiella et al. 2015; Hagelüken et al. 2016; Tansel 2017; Wilson et al. 2017) Publicly available information from the company (Rujanavech et al. 2016; Apple Inc. 2019a, b, 2020)
Cooling for superconducting magnets in MRI machines for medical imaging	Helium	Hospital or dedicated medical imaging service facility	Relevant literature (Epple et al. 1984; Nuttall et al. 2012; European Commission 2017, 2020; Butler 2017; U.S. Department of the Interior 2018; Anderson 2018; Lowe 2019; LBN Medical 2019; Rentz 2020; Kramer 2020) Publicly available information from an MRI machine manufacturer (GE Healthcare 2016) Personal communication with Ankesh Siddhantakar, helium industry expert, Toronto, Ontario, Canada

^*^Circularity strategies could also contribute towards supply security of other CRMs (e.g*.*, yttrium, along with rhenium, in jet engine turbine blades), but for simplicity, we focus on a single CRM (or group of CRMs, such as platinum group metals or rare earth elements)

^**^Due to the limited information on which to base our analysis of this case, we cannot highlight specific CRM(s)

## Analysis of case studies

### Rhenium in superalloys for jet engine and gas turbine components

Our first case concerns the use of rhenium as one of the key elements in single-crystal superalloys used in turbine blades and other components of the high-pressure and high-temperature section of modern jet engines and gas turbines. Rhenium is among the rarest and most geologically dispersed elements, with estimates of average crustal concentration ranging from 0.2 to 2 parts per billion (Kesieme et al. 2019). Like many CRMs, it is produced as a by-product of other commodities—in this case, as a by-product of molybdenum, some of which in turn is produced as a by-product of copper (Kesieme et al. 2019). Consequently, rhenium supply is dependent on this molybdenum production route; the economics are dominated by the markets for copper and molybdenum and not the price of rhenium (Ku and Hung 2014). Global rhenium production is on the order of 50 tons per year (USGS 2019, 2020, 2021). Rhenium is traded via supply contracts negotiated directly between value-chain participants.

Approximately 80% of global rhenium production is used for superalloys in various models of gas turbines and jet engines (Schulz DeYoung Seal Bradley. 2017; USGS 2020). Jet engines are estimated to represent the main market for superalloys, with several leading manufacturers: Cannon Muskegon, GE (essentially through its CFM International joint venture with Safran), Pratt & Whitney and Rolls Royce. In this case study, we take the perspective of the turbine manufacturer as the focal industrial actor in the rhenium value chain. The rhenium market experienced a well-documented supply crisis in the late 2000s, which was marked by a tenfold increase in pricing to over $10,000 per kilogram in 2008 (Kesieme et al. 2019). Turbine manufacturers responded with an urgent and deliberate approach. Along with research and development to minimize the rhenium content of superalloys (Fink et al. 2010), they adopted several circularity strategies to minimize material losses during manufacturing and to recover rhenium for end-of-life recycling (Konitzer et al. 2012). These circularity strategies significantly—albeit not fully—offset the increasing demand for primary rhenium supply to meet the needs of the growing jet engine market in the 2010s. This displacement of primary material demand can in turn reduce upward pressure on rhenium prices.

**Fig. 2:**
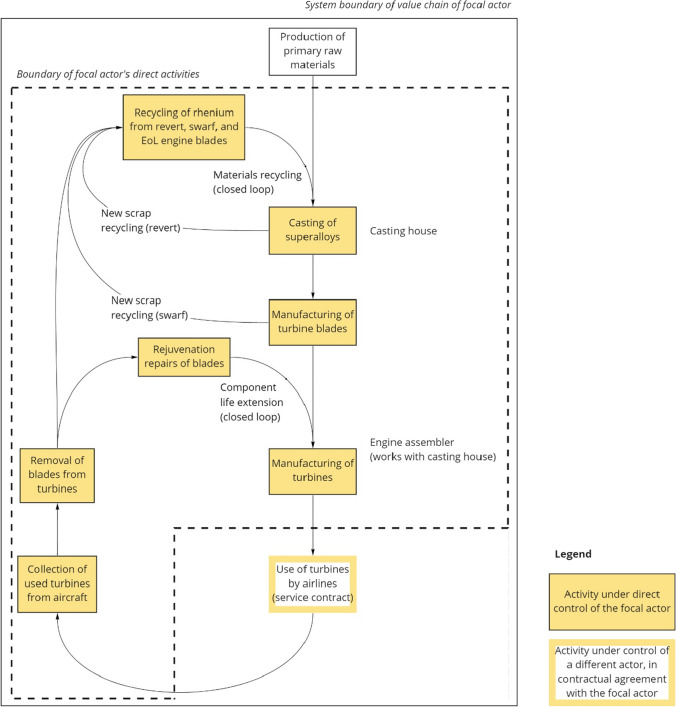
Circularity strategies for rhenium in superalloys for jet engine turbine blades. Our analysis takes the perspective of the turbine manufacturer as the focal industrial actor in the value chain. EoL, end-of-life

Complex business arrangements, including joint ventures, are sometimes used across the aerospace industry. In these cases, efforts to introduce circularity must be initiated by the entities that control the actual components; in this case study on rhenium use, this would include the blades and shrouds in the hot gas path of the aircraft engine. As illustrated in Fig. 2, the implemented circularity strategies include internal recycling of manufacturing scrap “revert” (from alloy casting) and “swarf” (from machining of cast components)—both of which we term *new scrap recycling*. Further circularity strategies include remanufacturing of blades in the form of “rejuvenation” repairs (which we term *component lifetime extension* (*closed loop*)), and ultimately recovery and recycling of rhenium from end-of-life engine blades (which we term *material recycling* (*closed loop*)) (Lee et al. 2008; Konitzer et al. 2012). Further details on recovery and recycling processes are elaborated by Dasan et al. (2011) and Srivastava et al. (2016, 2014).

The first circularity strategy—internal recycling of manufacturing scrap—fits naturally within the business imperative of manufacturing efficiency and value chain optimization. This strategy is facilitated by the closed-loop nature of the value chain wherein processing of the raw metal into the final form is largely maintained through vertically integrated operations involving internal manufacturing facilities and close business partners. The turbine manufacturers’ control of the rhenium material flows gives them visibility into different stages in the value chain. This, in turn, allows them to assess the cost-value trade-offs of different options and implement the most cost-effective processes.

Moreover, circularity strategies for turbine blades are a natural consequence of the “maintenance, repair, and overhaul” (MRO) business model used by the aviation industry. In this arrangement, the turbine manufacturer retains ownership of—and responsibility for—the turbine and the airline pays for the service provided by the turbine (Lee et al. 2008; Rodrigues Vieira and Lavorato Loures 2016; Rezaei Somarin et al. 2018). This business-to-business (B2B) arrangement allocates risk in a mutually favorable way, while providing a mechanism for in-service and end-of-life circularity strategies. As the turbine manufacturer maintains control of the rhenium stock, this model creates a strong opportunity for resource efficiency, especially when faced with severe price and/or supply shocks for primary materials. Within this model, careful labeling of turbine blades, with information identifying their material composition (including rhenium content), helps avoid “leakage” of rhenium that could occur if superalloys were to enter a mixed-alloy recycling stream (Konitzer et al. 2012).

### Platinum group metals in chemical processing catalysts

Our second case concerns the use of platinum group metals (PGMs), principally platinum, palladium, and rhodium, in industrial catalysts for chemical processing and oil refining^3^—not to be confused with catalysts used for automotive applications, for which the situation is different, as explained in subsequent paragraphs. The focal industrial actor in this case study is the catalyst user (i.e., chemical plant or oil refinery), which maintains ownership of the PGM stock and, therefore, is incentivized to implement cost-effective means to conserve material.

PGM spot prices are highly volatile with often extreme ranges within relatively short time periods^4^ (Hagelüken 2020; Johnson Matthey 2020). As with rhenium—the main CRM of interest in our first case study—PGMs are partly produced as by-products of other metals (Hagelüken and Meskers 2010; European Commission. 2020). Mining activities are highly concentrated, particularly in South Africa and Russia (Mudd et al. 2018; Hagelüken 2019, 2020; European Commission 2020; Johnson Matthey 2020; Yuan et al. 2020). PGMs are also difficult to substitute in catalytic applications (Hagelüken and Meskers 2010; Rasmussen et al. 2019; European Commission. 2020). Therefore, catalyst users (including chemical plants and oil refineries) have a strong interest in maintaining property of the PGMs contained in their catalysts (Hagelüken and Meskers 2010; Hagelüken 2012; Hagelüken et al. 2016).

**Fig. 3:**
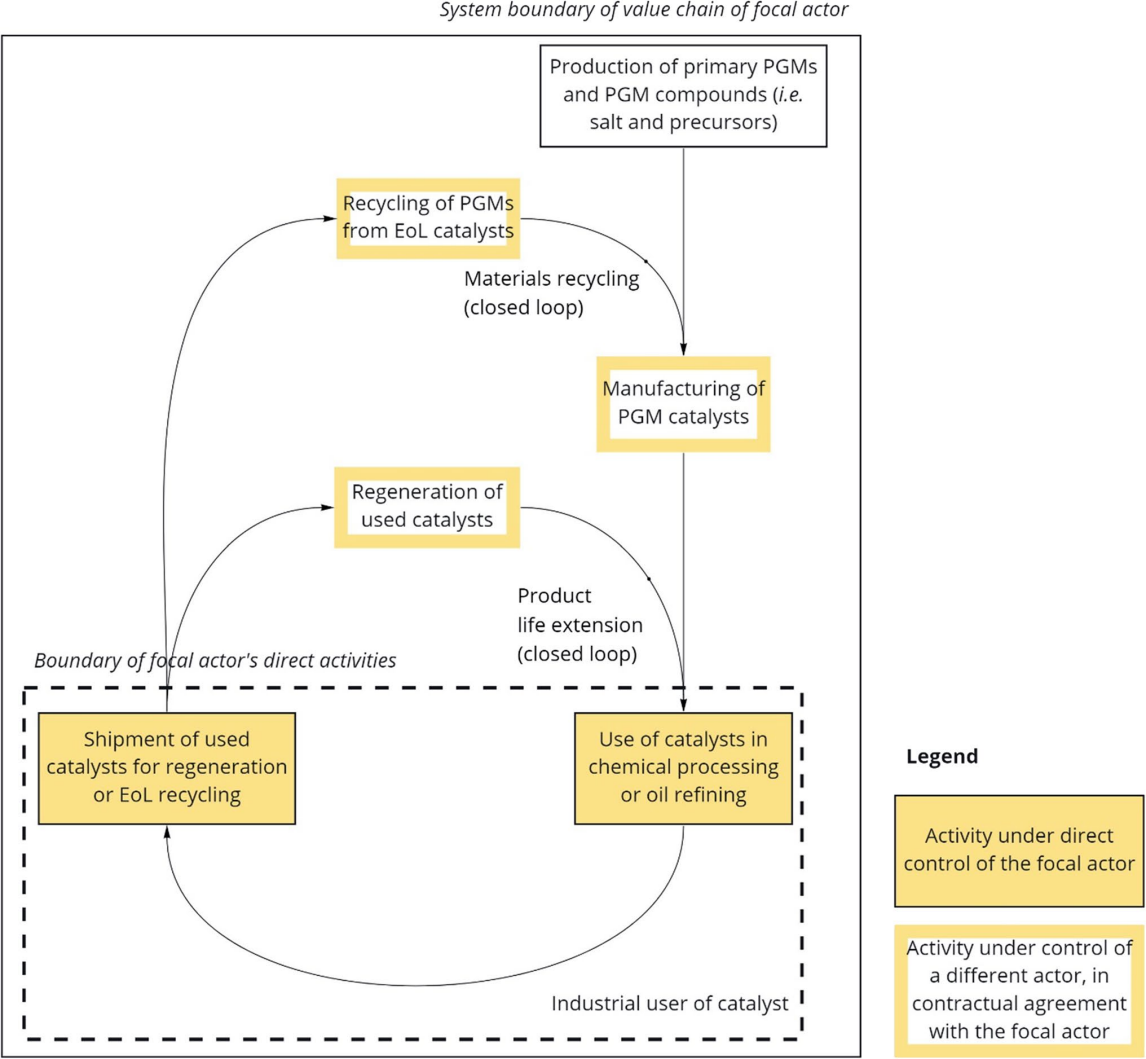
Circularity strategies for platinum group metals in industrial catalysts used in chemical processing and oil refining. Our analysis takes the perspective of the catalyst user (i.e., chemical plant or oil refinery) as the focal industrial actor in the value chain. PGM, platinum group metal, EoL, end-of-life

The simple value chain with few actors involved and relatively low transaction costs, combined with transparent information from all actors about the product, and the catalyst user’s control over the material at the end-of-life stage, create a favorable situation for circularity strategies. Like what was observed in our first case (i.e., rhenium-containing superalloys in jet engine turbine blades), circularity strategies—including product lifetime extension and end-of-life material recycling—are facilitated by an established B2B service model prevailing in the industrial catalysis market. As illustrated in Fig. 3, catalyst users maintain contracts with firms providing catalyst “regeneration” services (i.e., externally burning off carbon coatings) to extend the service lifetime of their catalysts (services which we term *product lifetime extension* (*closed loop*)). Ultimately, end-of-life catalysts are sent to firms specializing in PGM recycling (which we term *material recycling* (*closed loop*))—usually the same firms that manufacture the catalysts. Precise sampling and assaying of the PGM content in a specific catalyst shipment is conducted as the first step in the recycling service (Hagelüken and Meskers 2010). Under the recycling service contract, the catalyst user retains property of the contained PGMs—thus completing the closed-loop value chain.

By minimizing the need for primary raw materials (limited to “top-ups” compensating for small dissipative losses during use and in the regeneration and recycling processes), this closed-loop model strengthens supply security, improves overall economic performance, and insulates catalyst users from the volatility of PGM prices. In contrast, the automotive industry, which relies on PGM-containing catalysts (especially palladium and rhodium), to meet increasingly stringent vehicle emissions regulations, is more severely impacted due to key differences in the business model (i.e., B2B vs. B2C), value-chain structure, and “product mobility” (Hagelüken and Meskers 2010; Hagelüken et al. 2016; Rasmussen et al. 2019). Under a B2C model, automotive catalysts are widely dispersed among many (original and subsequent, mostly non-industrial) users and are often relocated—potentially crossing jurisdictional boundaries—with each change of ownership. Under a B2B model, chemical processing and oil refining catalysts are used in highly specialized applications involving a limited number of industrial actors, and the catalysts remain for their use in a fixed location during their entire service life. Consequently, the value chain is much more transparent and tightly controlled, especially at the product end-of-life stage. This model is so effective that, in aggregated production and use data, the industrial catalysis applications of PGMs appear much smaller than they actually are (i.e., the demand for primary production of PGMs in these applications is minimized due to the catalyst lifetime extension and end-of-life recycling processes). A global material flow analysis by Rasmussen et al. (2019) indicates that chemical processing and oil refining use on the order of 59 t and 32 t of platinum per year, respectively, of which closed-loop recycling fulfills 72% and 83%, respectively.

### Permanent magnets in computer hard disk drives

Our third case concerns the use of the rare earth elements (REEs) neodymium and dysprosium in neodymium-iron-boron (NdFeB) permanent magnets for computer hard disk drives (HDDs). As of 2014, HDDs accounted for about 16% of the total demand for NdFeB magnets by value (Constantinides 2016). This fraction has subsequently been declining due to the growing uptake of solid-state drives (SSDs), which provide an alternative to HDDs that do not require REEs. The focal industrial actor in this case is Hitachi Group, a manufacturer of NdFeB magnets with no domestic Japanese REE supply source, which has created a mechanism to collect the product at end-of-life and recover the CRMs of interest (Baba et al. 2013; Nemoto et al. 2019). Under the National Permit System of the Japanese Ministry of the Environment, Hitachi Group companies and regional affiliates manage a nationwide collection of waste electrical and electronic equipment (WEEE)—including, but not limited to, personal computers, servers, automated teller machines (ATMs), and HDDs—for disassembly and material recovery in facilities affiliated with Hitachi Group (Nemoto et al. 2019; Harada and Nemoto 2020). To limit the scope of our analysis, we focus on recovery and recycling of permanent magnets from HDDs, which is particularly noteworthy given the technical and economic challenges involved (Binnemans et al. 2013; Sprecher et al. 2014; Habib et al. 2015; Yang et al. 2017; Lixandru et al. 2017), and how Hitachi Group responded to them.

**Fig. 4:**
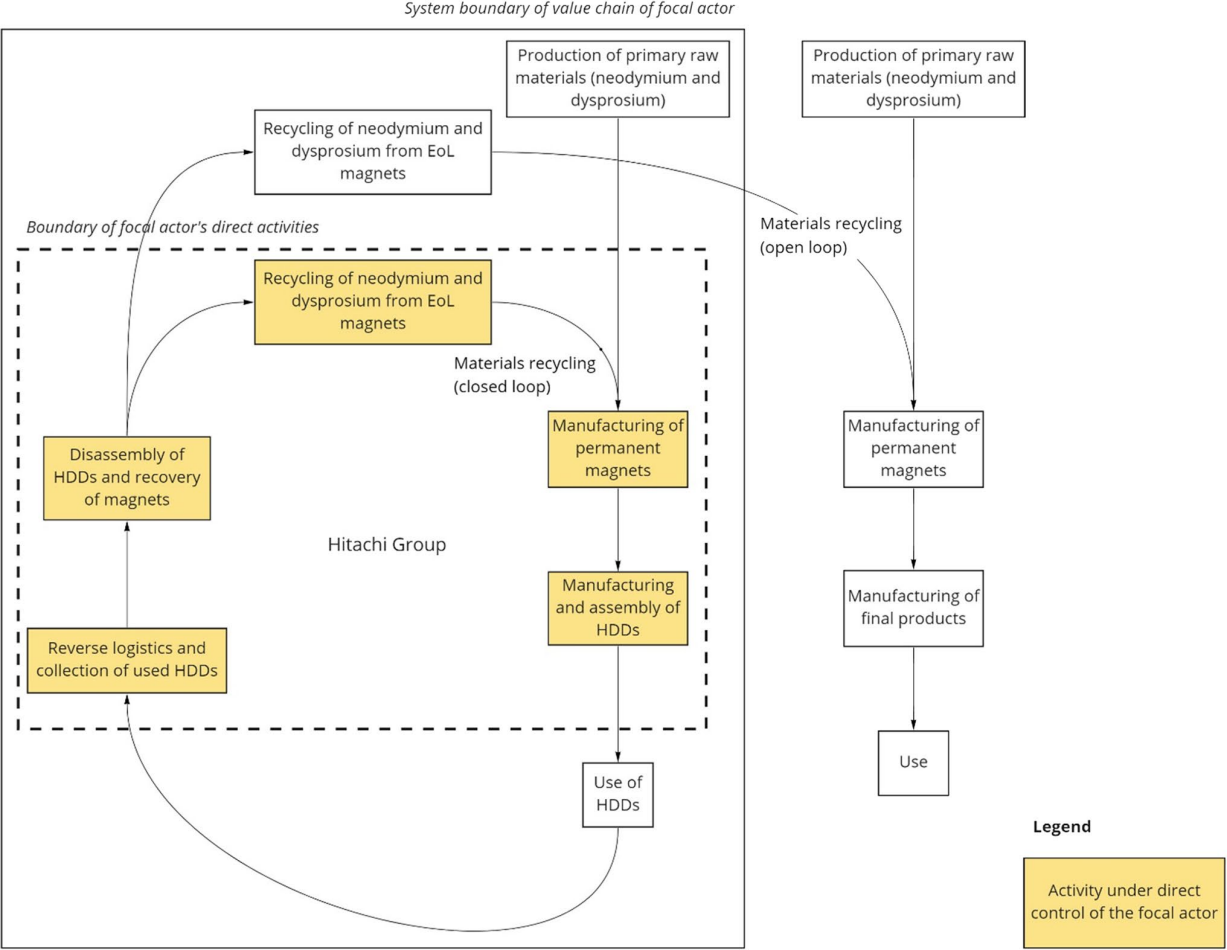
Circularity strategies for neodymium and dysprosium used in permanent magnets for computer hard disk drives (HDDs). Our analysis takes the perspective of Hitachi Group as the focal industrial actor in the value chain. EoL, end-of-life

As illustrated in Fig. 4, the collected HDDs are disassembled using specialized equipment designed by Hitachi Group. First, the HDDs are placed in a machine with a rotating drum that generates repeated shocks and vibrations that loosen the HDD’s mechanical fasteners and ultimately separate the component parts (Nemoto et al. 2019). This machine enables an order of magnitude improvement in the efficiency of the disassembly process: Whereas manual disassembly can be done at a rate of about 10–12 HDDs per worker per hour, the automated process can be done at a rate of about 140 HDDs per worker per hour (Nemoto et al. 2019). Specially designed through holes in the drum of the disassembly machine allow voice coil motors (VCMs)—containing the permanent magnets—to pass through with minimal damage, thus maximizing material recovery potential (Nemoto et al. 2019). Subsequently, another machine separates scrap materials (including ferrous metals, aluminum, glass, and circuit board fragments containing precious metals) from the HDD components (Baba et al. 2013). A third machine recovers the permanent magnets from the VCMs that were in turn recovered from the HDD disassembly machine (Baba et al. 2013). Finally, the recovered magnets are sent to magnet manufacturers, both within and outside of Hitachi Group (i.e., a mix of open and closed material loops), where (at least in the case of Hitachi Group magnet manufacturers) neodymium and dysprosium are extracted and recycled into new magnets.

In this case, the focal actor expanded its role in the value chain to include collection and disassembly of products at their end-of-life. Technical advances reducing the cost of these steps were essential in improving the economics of this circularity strategy. In contrast to our first two cases, which involved B2B arrangements, HDDs are sold in a business-to-consumer (B2C) market—making it more challenging to coordinate logistics for the recovery process. The added cost can be a barrier that hinders widespread adoption of recycling (Hagelüken and Meskers 2010). Here, government intervention, through the passage of Japanese laws such as the *Act on Recycling of Specified Kinds of Home Appliances* and the *Basic Act on Establishing a Sound Material-Cycle Society*, was an enabling factor providing incentives via regulation and subsidies. Fueled by these incentives, the company’s circularity strategies for rare earth magnets have in the meantime also been beneficial in terms of cost efficiency and supply security. At the time of writing, material recovery and recycling fulfill about 10% of Hitachi Group’s total demand for rare earth magnets (Nemoto et al. 2019).

### Consumer electronics

Our fourth case extends the lessons from Hitachi Group to another industrial actor in the consumer electronics industry—Apple Inc. We have limited information on which to base our analysis of this case—principally from the company’s website, reports, and press releases (Rujanavech et al. 2016; Apple Inc. 2019a, b, 2020). Nonetheless, this case is illustrative of the differences between B2B and B2C value chains, and of some ways in which the latter could mimic some of the advantageous elements of the former—particularly concerning transparency and control over CRMs from the perspective of the focal actor (as seen in our previous cases and elaborated in our discussion).

With the aspiration of “using only recycled and renewable material in [its] products,” the company has developed “material impact profiles” (MIPs)—incorporating assessments of environmental and social impacts, along with risks to supply security—for 45 materials commonly used in consumer electronics (Apple Inc. 2019a). The MIP results, weighted by the quantities of the materials used in Apple products, informed the creation of material-specific working groups—comprising experts from engineering, procurement, operations, supplier responsibility, and environmental teams—tasked with “closing the loop” for each material (Apple Inc. 2019a). A highly publicized initiative (with a press release dated April 18, 2019) towards Apple’s goal of closing material loops is the company’s development of specialized robots for disassembly of mobile phones (Rujanavech et al. 2016; Apple Inc. 2019b). The company claims that its second-generation disassembly robot can disassemble 1.2 million devices (with the ability to process 15 different phone models) per year (Apple Inc. 2019b). It is not clear how many end-of-life products actually have been dismantled (noting that the claimed disassembly capacity of 1.2 million mobile phones per year accounts for less than 1% of annual sales of these devices), or which materials are recovered (aside from cobalt in batteries, as highlighted in the aforementioned press release). Nor is it clear what proportion of the recovered materials are recycled back into Apple products (i.e., forming a closed-loop value chain), and if/when the disassembly program will be expanded to other Apple products (e.g., laptop computers, tablets, and watches).

**Fig. 5:**
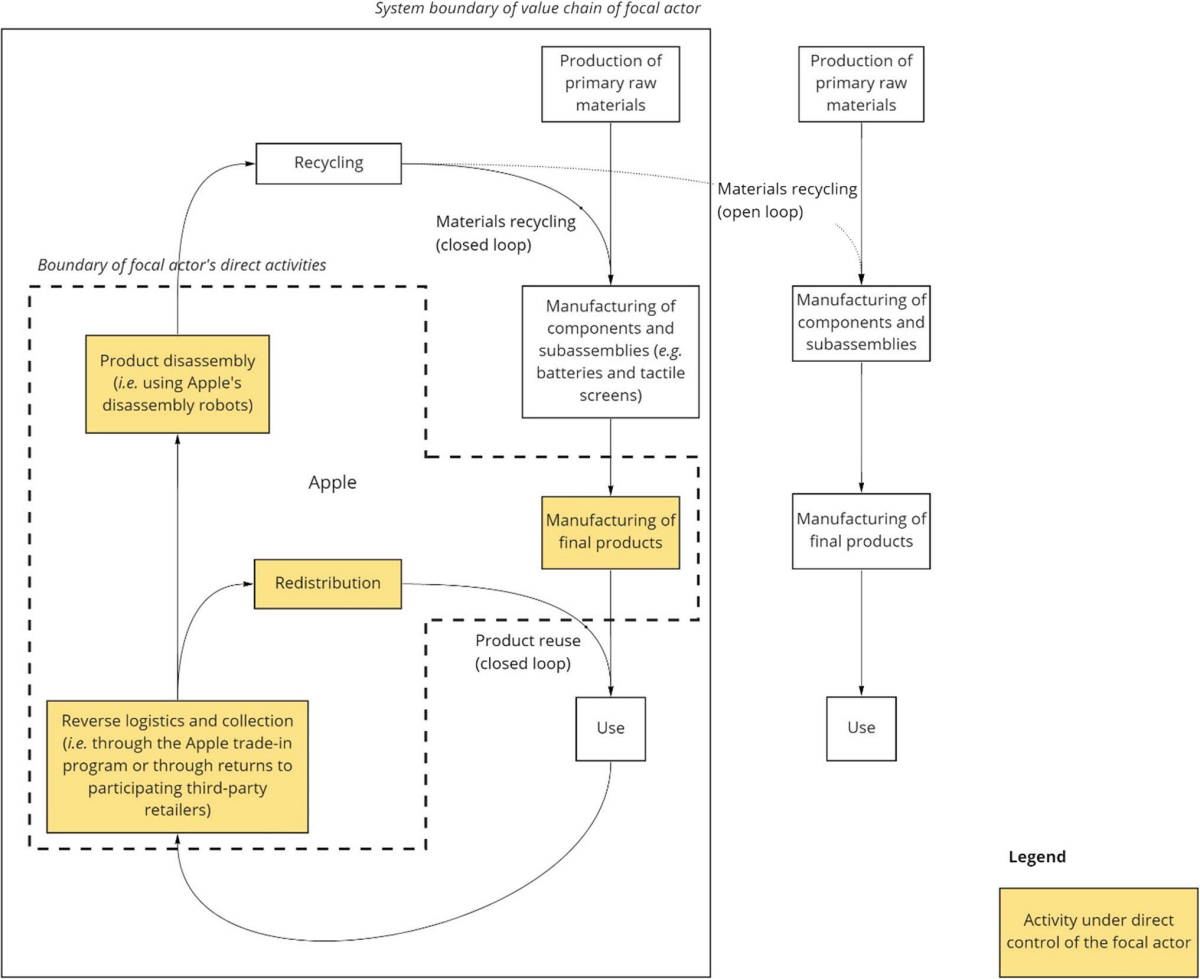
Circularity strategies for Apple’s consumer electronics products. Our analysis takes the perspective of Apple Inc. as the focal industrial actor in the value chain. The open-loop recycling of materials is illustrated as a dotted arrow to indicate the uncertainty over the proportion of open-loop vs. closed-loop material recycling

The success of product disassembly and material recycling programs for consumer electronics hinges on the collection of end-of-life products in the first stage of the recycling value chain (Hagelüken and Meskers 2010; Tanskanen 2013; Cucchiella et al. 2015; Hagelüken et al. 2016; Tansel 2017). As we noted in previous case studies, this is more challenging in a B2C value chain than in a B2B value chain. Considering precious metals, for example, end-of-life recycling rates from consumer electronics are typically less than 25%, though recycling rates over 95% are technically feasible (Hagelüken et al. 2016). As illustrated in Fig. 5, Apple has collection programs through its own branded retail locations as well as through third-party retailers. Through the Apple trade-in program, owners of Apple products (including phones and other devices as listed on the company’s website (Apple Inc. 2020)) can return the product via mail or at one of the company’s own retail locations. Depending on the condition of the product, it will either be resold (in exchange for a gift card or credit towards the purchase of a new Apple product) or sent for disassembly and material recovery. As announced in the previously cited press release from April 2019, Apple products can also be returned to participating third-party retailers, including Best Buy locations in the U.S. and KPN retail locations in the Netherlands (Apple Inc. 2019b).

Though Apple’s circularity strategies are in their infancy, the convenience and legitimacy of the company’s collection programs—operated directly through Apple stores or through third-party retailers—could encourage consumers to return their old Apple products rather than keeping them in “hibernation” (i.e., in storage but not in use—see Nokia Corp. (2008) and Wilson et al. (2017) for more information on this phenomenon), especially when given financial incentives. By addressing the problem of hibernating stocks, these incentives could help increase material recovery and recycling rates from consumer electronics, but so far there is not a strong instrument like a deposit or lease fee gearing consumer behavior towards product return. With bound monetary incentives like gift cards or trade-in credits—which also serve the company by binding consumers—the incentives to return the product in a timely manner (e.g., when buying a new product) might increase compared to cash returns, thus further approximating B2B mechanisms.

### Helium in MRI machines for medical imaging

Our fifth and final case concerns the use of liquid helium as a cryogen to cool superconducting magnets in MRI machines for medical imaging. Despite being subject to longstanding concerns over resource control and ease of access (Epple et al. 1984; Nuttall Clarke Glowacki. 2012; Butler 2017)—and being recognized as a CRM and a strategic material by governments (e.g*.*, by the European Commission (2017)^5^ and the U.S. (2018), helium (along with other gases used for industrial and medical applications) is still less widely discussed in the criticality literature compared to critical metals—despite the fact that almost all uses are dissipative in nature, and that helium has exceptional attributes, including cryogenic properties, that can preclude substitution. In the context of medical imaging, liquid helium is used in MRI machines because helium has the lowest boiling point of any element. Medical imaging is the largest cryogenic application, accounting for about 20% of global helium demand (Nuttall Clarke Glowacki. 2012; Anderson 2018). Manufacturing of MRI systems is dominated by a few large brands (including General Electric, Siemens, Philips, and Toshiba), with what we estimate to be a total of about 15,000 machines in use worldwide.

In this case study, we take the perspective of an industrial actor in a sector that, prior to the COVID-19 pandemic, was not widely discussed in relation to material criticality. Specifically, the focal industrial actor is a large modern hospital or other medical imaging facility that may operate several MRI machines in parallel, providing efficient 24-h imaging services. Medical imaging facilities maintain contracts with MRI vendors that include maintenance and helium provisions from local suppliers. Conventional MRI machines contain on the order of 1000–3000 L of liquid helium coolant, depending on model and vintage. However, given its small particle diameter and mass, liquid helium readily leaks and consequently frequent top-ups to magnets are needed, presenting both an operating cost and a resource loss. Normal helium losses often run up to 50% per year (Rentz 2020), and helium consumption can cost a facility from $25,000 to $100,000 annually (estimate based on Lowe (2019)). Moreover, helium supply is relatively uneven and prices fluctuate over time and by region (LBN Medical 2019; Kramer 2020).

**Fig. 6:**
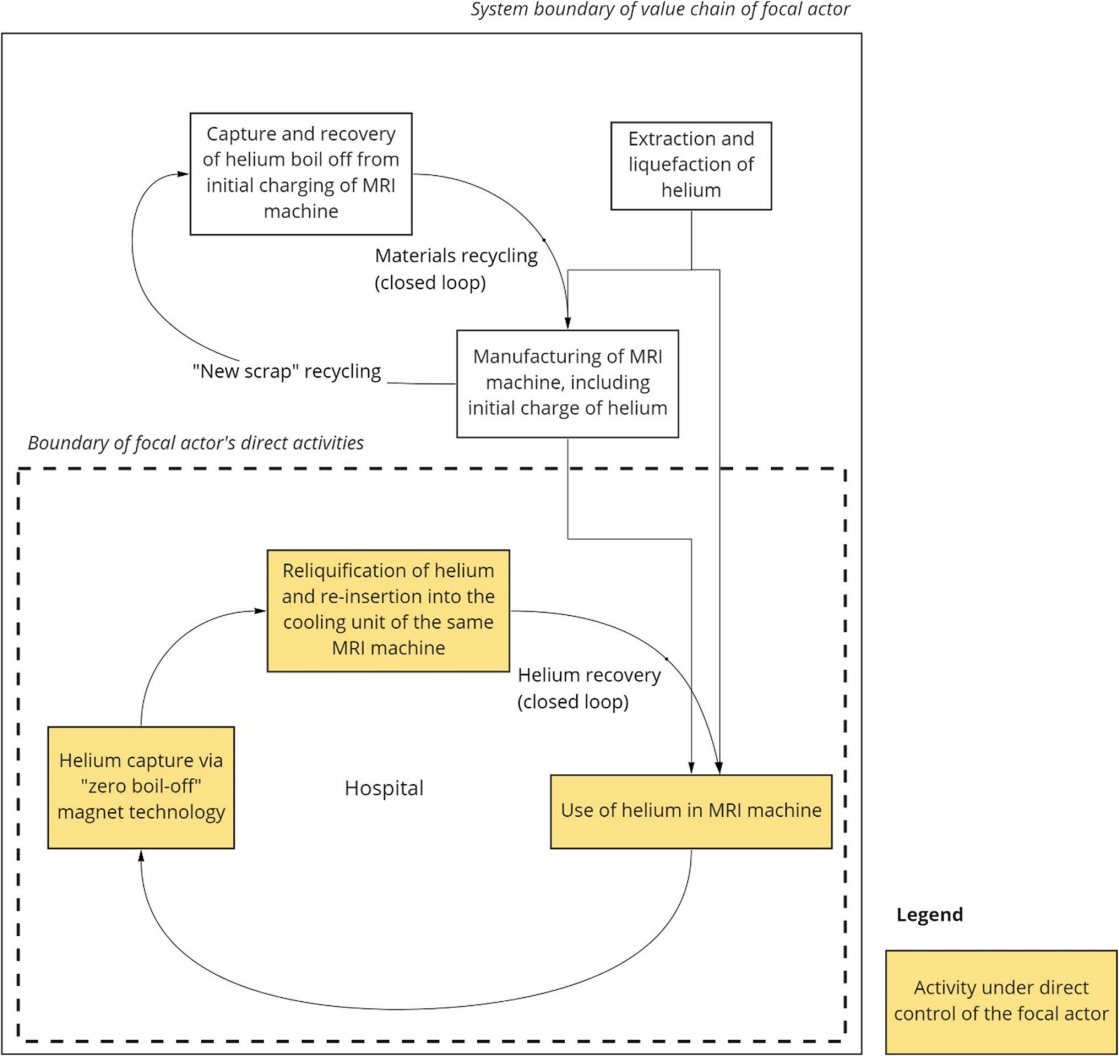
Internal reuse of cryogenic helium in “zero boil-off” MRI machines for medical imaging. Our analysis takes the perspective of the medical imaging facility as the focal industrial actor in the value chain

Around 2015, new MRI machines were developed that provide internal reuse of helium, thus extending the lifetime of the helium resource and drastically reducing specific helium consumption. This new “zero boil-off” magnet technology (GE Healthcare 2016) operates at 4 K and, as illustrated in Fig. 6, includes a helium capture and compressor system that re-liquifies the helium gas back into the cooling unit. Input of helium is still required for the initial charge, which uses thousands of liters and results in initial boil-off, but MRI manufacturers can capture this helium and return it to a central facility for liquefaction. Otherwise, helium losses are mostly limited to times of MRI machine maintenance or power loss. Although the design and manufacturing of MRI machines, including those incorporating zero boil-off technology, is beyond the direct control of medical imaging facilities, the advent of this new technology nonetheless helps maintain a closed-loop system that insulates medical imaging facilities from helium supply insecurity and price fluctuation. However, given the substantial capital investment and long lifetimes of MRI machines, turnover of the technology may take more than 20 years.

## Discussion

Together, our five case studies provide tangible examples of different circularity strategies (including material recycling, product reuse, and various forms of product or component lifetime extension) implemented for specific applications of CRMs (including precious metals, rare earth elements, and a noble gas) by specific industrial actors across a range of industries (including aviation, chemical processing, consumer electronics, and healthcare). While these cases are neither representative of all possible scenarios nor equally mature or well documented, they nonetheless demonstrate the value of our adaptation of the Resource States framework developed by Blomsma and Tennant (2020), which provides a contextualized approach in examining company circularity strategies for CRMs, recognizing the nuances of both the material criticality and circular economy concepts. Furthermore, drawing from our case studies, three broader observations can be made about company circularity strategies for CRMs. The first concerns the motivations of industrial actors for implementing circularity strategies. The second concerns the role of business models and value-chain structure in enabling and facilitating circularity strategies, and the third concerns the significance of our distinction between open-loop and closed-loop circularity.

To support a pathway to sustainable development, circularity strategies need to provide a benefit for the companies that implement them. Our case studies demonstrate that there are several factors that can motivate industrial actors to implement circularity strategies for CRMs. These include common business motivations like cost savings (i.e., improved manufacturing and operational efficiency), along with reduced exposure to material supply insecurity, to price volatility, or to the risk of regulatory constraints. However, these motivations may not be sufficient if the business benefit of circularity strategies (i.e., from the value of recovered materials) is not enough to offset the cost of implementing them. It is important to note that in our third case—concerning Hitachi’s circularity strategies for permanent magnets in HDDs—the implemented circularity strategies were made economically viable by the development of new technology and equipment for automated disassembly of HDDs, which in turn was supported through government intervention.

Government intervention could be aimed at alleviating supply risks in the short term or creating more sustainable supply structures in the long term. Financial support, via tax rebates and subsidies, may contribute to the economic viability of circularity strategies in the short term. However, such interventions may appear costly if the economic viability of the circularity strategies remains dependent on uncertain market conditions. Regulation alone could discourage companies from developing their activities nationally, if the regulations create an uneven playing field internationally. A combination of government interventions could help create a stable demand for recycled materials, regardless of fluctuating market prices of primary materials (e.g*.*, via mandatory recycling rates or recycled contents). Financial aid could overcome investment thresholds when short-term market outlooks make circularity strategies economically unfavorable, thus allowing industries to reach economies of scale. Successful government intervention hence requires a long-term vision and engagement with national industries.

Besides such policy incentives, other forms of external stakeholder pressure—such as the imperative of protecting the reputations of iconic and valuable brands (like Apple in the consumer electronics industry) from an environmental, social, and governance (ESG) standpoint—can also motivate circularity strategies (and marketing of such strategies).

Regarding the role of business models and value-chain structure in enabling and facilitating circularity strategies, our case studies suggest that B2B models may be more conducive to circularity than B2C models. Our case studies demonstrate that in a B2B environment, property rights are well defined and held by a small number of actors along the product life cycle. Consequently, the economic benefits of B2B relationships are clearly identifiable and transaction costs are lower than in B2C relationships (Hoejmose et al. 2012). Lower transaction costs make it easier for market actors to internalize the external costs (i.e., with respect to ESG concerns) of implementing circularity strategies. Moreover, well-defined property rights facilitate rational economic decision-making considering the costs and benefits of circularity strategies such as capital investments in capacity for product disassembly and material recovery, weighed against the benefits of mitigating material supply shortages and price volatility.

Another observation from our case studies is that B2B value chains tend to be more transparent for the focal industrial actors and can therefore be more tightly controlled by them,^6^ especially where the business model is structured around long-term service contracts. This is in line with the observation of Elia et al. (2020) that there is a correlation between the level of “supply-chain integration” and the number and type of implemented circularity strategies in the supply chain. Another factor could be that consumers are less motivated to engage in circularity strategies due to the (at least perceived) difficulty and inconvenience of the necessary actions (e.g*.*, directing end-of-life electronics products and components—such as batteries—to appropriate material recovery and recycling channels) and their limited capacity for benefiting from the value of CRM recovery. As the recoverable material value in, e.g*.*, a single electronic device is low (while overall hundreds of millions of devices globally sold have a big impact on CRM demand), other incentives like deposit systems or lease fees would be needed to effectively pull such consumer products into recycling or other circularity strategies. The combined effect of these factors results in B2B models having fewer actors who can better internalize external costs and can act upon better information, with lower transaction costs and stronger business incentives.

As an illustrative example, the global EoL recycling rate of PGMs from automotive catalysts (i.e., in a B2C ownership model) is significantly lower than for chemical processing catalysts (i.e., in a B2B service model), despite the intrinsic value of the contained PGMs being of comparable magnitude. Recycling technologies are very mature in both cases, making recycling highly attractive from an economic standpoint. In addition to the effects of dispersed use of automotive catalysts, multiple ownership, and relocation, the value of the catalyst in this application is concealed in the value of the automobile. End-of-life vehicles (ELVs) from Europe and other industrialized countries are widely exported to developing and transitional countries. In this case, the residual value of the vehicle outweighs the value of the PGMs in the catalysts (which remains embedded in the vehicle). Many of the importing countries lack an appropriate recycling infrastructure and technical supervision for vehicles in use. Hence, during use in these countries and at vehicle end-of-life, there is substantial leakage of PGMs from potential material recovery loops. The same is often true for actors involved in trading catalysts dismantled from such vehicles, some of which also derive from ELV exports. The vehicle owners themselves lack information about the value of the catalyst and cannot play an active role in closing the loop, while the original equipment manufacturer (OEM) has no property rights and usually also no knowledge about the final whereabouts of the vehicle at end-of-life. Shifting to a more service-based business model could provide a promising avenue for circularity strategies.

We do not intend to suggest that circularity strategies for CRMs can never work in B2C models. The Apple case, for example, shows some promise due to key aspects, like the product trade-in program, which mimic some of the advantageous elements of a B2B model (particularly by incentivizing consumer actions and improving value-chain transparency). The Hitachi case (the “Permanent magnets in computer hard disk drives” section) demonstrates that a synergy of policy and technological development can enable increased circularity in a B2C context and provide concrete benefits in terms of supply security to the focal actor, though to a lesser extent than observed in the B2B examples (the “Rhenium in superalloys for jet engine and gas turbine components” and “Platinum group metals in chemical processing catalysts” sections).

It is also worth highlighting that our adaptation of the Blomsma and Tennant (2020) framework distinguishes between *open-loop* circularity (i.e., in which material flows cross the system boundary of the focal actor’s value chain) and *closed-loop* circularity (i.e., in which the material flows are contained within the system boundary). This is an important distinction from the perspective of material criticality, where the primary objective of circularity strategies—as seen in our cases (especially the first two cases)—is to use material recovery loops to minimize the need for primary material inputs and thereby mitigate the impacts of supply shortages and/or price spikes of those materials. Suppose for example that turbine manufacturers were to send end-of-life turbine blades through a mixed recycling stream, and the rhenium recovered from the blades was to be used in other applications by different actors (i.e., in what we would consider to be an open-loop). Depending on the perspective taken, this scenario could still be considered an example of circularity, and it could even be described as “closed-loop” (e.g*.*, from the perspective of a national or regional economy, or from the perspective of an industrial ecologist (see Table 1)). Yet, from the perspective of the turbine manufacturer, this circularity strategy would not provide the same benefits (in terms of mitigating material criticality) as recycling the rhenium back into new turbine blades. It is also important to note, however, that although such closed-loop circularity can bring benefits in terms of supply security, it does not necessarily coincide with financial or environmental optimization. Additional efforts during collection may be necessary to return the material to the country in which the original product was produced, compared to valorizing the material locally. Also, Geyer et al. (2016) argue that from an environmental standpoint the application of recycled material is irrelevant; the parameter that influences the environmental performance of the circularity strategy is the specific primary material that is *substituted* (and its accompanying environmental impacts), regardless of by whom this substitution is applied. In other words, open-loop recycling as observed in, for example, the Hitachi case (the “Permanent magnets in computer hard disk drives” section) might be as environmentally beneficial and equally cost-effective as closed-loop recycling, although the open-loop has a limited supply risk mitigation potential.

Finally, we acknowledge the unavoidable problem of data requirements for every circularity strategy that is at least partially “open loop” from a business perspective. Novel approaches towards data sharing without compromising company confidentiality could help in improving the knowledge base, in particular regarding the presently limited data on the composition of products and components, thus supporting policy makers in setting the framework conditions for effective recovery strategies and aiding industry in implementing them.

## Conclusions and outlook

Material criticality is likely to continue being of increasing concern over the next decades, given the widespread changes to energy generation and storage infrastructure needed to support the transition to a low-carbon economy, the development of transitional countries, and the pace of advancing technologies—especially in e-mobility and digitalization—enabled by specialized material sets. Our case studies suggest that the motivations of industrial actors in the adoption of circularity strategies, and the design of business models conducive to these strategies, are key areas for future research on the link between the CE and material criticality. In particular, future research could further investigate the factors affecting the technical and economic feasibility of different circularity strategies, and how otherwise well-designed circularity strategies can be impacted by external factors beyond the control of a single industrial actor—such as changes in technology, consumer preferences, and market conditions (Ku et al. 2018). For example, a technological shift towards light-emitting diode (LED) lighting, which dramatically reduced the use of rare earth phosphors in this application, had profound market implications for these materials—including a reduction in closed-loop recycling (Ku et al. 2015). We also note that companies comprise different functions (e.g*.*, sourcing, operations, engineering, and compliance/legal/sustainability) that have different interests, authority, time horizons, and risk tolerance. The interplay between these functions is important to the design of business models supportive of circularity strategies and therefore needs further exploration in future studies. Another important element is the role of the legislative and regulatory environment in motivating—or possibly impeding—circularity strategies. More broadly, different aspects of state policy might be aligned to the interests of different functions within a business. Specifically, economic incentives would appeal to sourcing and finance functions which are driven by bottom-line considerations, whereas regulatory guidelines impact compliance functions. In this regard, a “carrot-and-stick” approach to designing policy initiatives could be more effective than an approach that treats businesses as monolithic, purely rational economic actors.

Given the nuances of material criticality, solutions to CRM problems must be tailored to each situation. Our work suggests that circularity strategies can be a valuable option—alongside other strategies like value-chain diversification and material substitution—for addressing material criticality. Recognizing the perspectives of industrial actors and systematically evaluating the specific challenges and opportunities in different sectors can help identify ways to effectively implement circularity strategies for CRMs.

## Supplementary Information

Below is the link to the electronic supplementary material.

Supplementary file1 (DOCX 373 KB)

## Footnotes

1 Though for simplicity we refer to our framework as a “*company*-level CE framework,” we intend for it to be broadly applicable to any company, organization, or institution in any industry or sector.

2 In this article, we consider “circularity strategies” to be synonymous with “looping” strategies (i.e*.*, in which material flows are either contained within the system boundary of the focal actor’s value-chain, or in which material outflows from the focal actor’s value-chain become inflows to another industrial actor’s value-chain). We do not consider other resource efficiency strategies, like “dematerialization,” increased product longevity, or materials substitution as constituting *circularity* strategies in this regard.

3 This case refers to the bulk of heterogeneous and homogeneous process catalysts used, *e.g.*, for naphtha reforming or catalytic cracking in oil refining or for numerous catalytic syntheses in bulk and fine chemistry, which remain in the chemical reactor and hence can be recycled at their end of life. However, some applications exist where the PGMs are consumed into the product and hence are dissipated, making them unavailable for PGM recycling (Hagelüken 2008).

4 For example, the price of rhodium increased from 2500 to over 30,000 $/oz between early 2019 and April 2021, the palladium price increased by a factor of 2.5 in the same period, and platinum increased by 40%, with significant fluctuations during this period. This market volatility underscores why maintaining the property of the PGMs along several catalyst life cycles can bring clear economic benefits to industrial users (Hagelüken 2020).

5 Helium was dropped from the EU CRM list in the 2020 update (European Commission 2020).

6 B2B loops can also be much better controlled by supervising authorities, thereby supporting the internalization of external (societal) costs. A (larger) industrial actor in a country with a strong policy environment needs to follow the rules around process emissions, workplace safety, etc., and is regularly controlled by authorities (and civil society). This is much more difficult in a B2C environment, both at the level of consumers and of traders and often small/dispersed/informal actors in product EoL management.
