# Supplementary material for: The role of industrial actors in the circular economy for critical raw materials: a framework with case studies across a range of industries
Source: Miner Econ. 2022 Feb 21;36(2):301–19. doi: 10.1007/s13563-022-00304-8 (PMC8860293; doi:10.1007/s13563-022-00304-8)
Supplement: Supplementary file 1 — Supplementary file1 (DOCX 373 KB) [file 13563_2022_304_MOESM1_ESM.docx]

**Supplementary Information: Composition of the framework and case study selection**

*The role of industrial actors in the circular economy for critical raw materials: A framework with case studies across a range of industries*

We developed our framework and case studies through an iterative process beginning with an IRTC meeting at the EU Raw Materials Week in Brussels in November 2019, after having discussed circularity strategies for CRMs at two previous IRTC Round Tables (reports available at <https://irtc.info/wp-content/uploads/2019/05/Summary-Round-Table-Ecobalance-.pdf> and <https://irtc.info/wp-content/uploads/2019/11/Summary-IRTC-Round-Table-Beijing-1.pdf>). At the meeting, 29 Consortium and industry focus group members were present, with two of them participating online. The geographic distribution covered the EU, Switzerland, Norway, the USA, Canada, Korea, Japan, Australia, and China.

At the meeting, an initial framework was developed to classify circularity strategies for CRMs. This framework, loosely based on the work of Bocken et al. (2016) and the Ellen MacArthur Foundation (2013), comprised a two-dimensional matrix reflecting the types of risks and the circularity strategies concerning the specific case.

Table 1: Initial table for analyzing case characteristics, as developed at the IRTC Meeting in Brussels, November 19, 2019.

|  |  | **Design strategies** | | | **Longevity** | | **Looping strategies** | | | | | | | | |
| --- | --- | --- | --- | --- | --- | --- | --- | --- | --- | --- | --- | --- | --- | --- | --- |
|  |  | System design / Business model | Product design / Technology choice | Material selection/ substitution | Product longevity / Durability | Repair | Reuse | Remanufacture / Refurbishment | | | | Recycling | | | |
| **Supply** | Constraint in choice of material supply |  | |  | |  | | |  |  |  | |  |  |  |
| **Risks** | Political risk at source |  | |  | |  | | |  |  |  | |  |  |  |
|  | Geological risk at source |  | |  | |  | | |  |  |  | |  |  |  |
|  | Environmental risks at source |  | |  | |  | | |  |  |  | |  |  |  |
|  | Social risks at source |  | |  | |  | | |  |  |  | |  |  |  |
|  | Process limitations at source |  | |  | |  | | |  |  |  | |  |  |  |
| **Demand** | Constraint in quantity demanded |  | |  | |  | | |  |  |  | |  |  |  |

This initial framework was discussed in various online meetings of the core authoring group (Alexander Cimprich, Steven B. Young, Dieuwertje Schrijvers, Anthony Ku, Christian Hagelüken, and Alessandra Hool). Finally, it was decided that the purpose of the paper – highlighting the role of ownership across the value-chain for circularity strategies – could be best reflected by a graphical depiction of the product system that was able to highlight the system boundaries, which emerged to be crucial for distinguishing between different circularity strategies. Therefore, we developed a framework that builds upon the “resource states” framework developed by Blomsma and Tennant (2020), structured around the flows of CRMs through the value-chain (Figure 2).

**
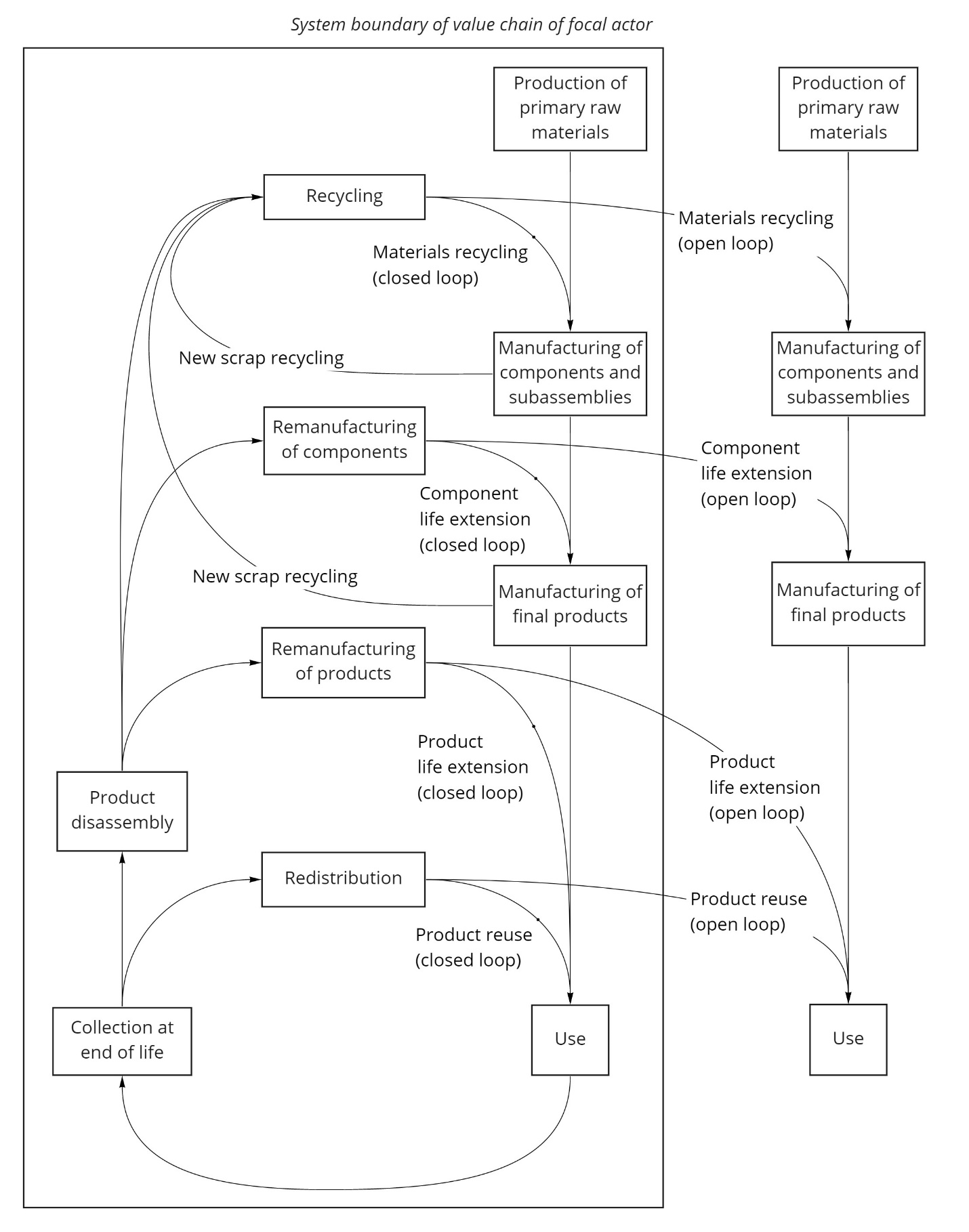
**

Figure 2: Value-chain framework for examining circularity strategies for critical raw materials from the perspective of a focal industrial actor (adapted from Blomsma and Tennant (2020)).

At the initial meeting and during the Raw Materials Week, all Consortium and industry focus group members were asked to suggest potential case studies of circularity strategies for CRMs. Subsequently, another call for cases was made via e-mail, and Consortium members were asked to fill in case studies and their parameters in a survey via Google forms. The survey comprised the following questions:

- Who is(are) the stakeholder(s) involved? Which industries, companies, etc. (could be multiple stakeholders, e.g., at different levels in the supply-chain)?
- Who is the best contact person to provide additional information regarding this case study? Please provide sufficient contact info (e.g., email address).
- What raw material(s) were of concern to the stakeholder(s) in this case?
- Why were these raw materials(s) of concern to the stakeholder(s)?
- What strategy(ies) was(were) implemented by the stakeholder(s)? Select all that apply.
  - Diversification of suppliers
  - Localization of supply-chains / domestic production / vertical integration
  - Development of new extraction and/or processing technologies
  - Product (re)design for circularity (e.g., design for longevity, reuse, repair, remanufacturing, and/or recycling)
  - Improved efficiency in manufacturing (e.g., reducing yield losses, reusing or recycling scrap)
  - Product reuse / repurposing / cascaded use
  - Product repairs and/or upgrades
  - Product remanufacturing
  - End-of-life recycling
  - Redesign of business models
  - Other:
- What was(were) the motivation(s) of the stakeholder(s) for implementing the above strategy(ies)?
- What was(were) the outcome(s) for the stakeholder(s)?
- When did the events in this case study take place?
- Where did the events take place? Where is(are) the stakeholder(s) located?
- What other thoughts or insights can you share in relation to this case study?
- Where can we go to find more information? Please provide references to relevant publications, documents, etc.

Based on this internal survey, we compiled an initial list of 14 potential case studies:

1. High-temperature superalloys in jet engine turbine blades
2. Reusing lithium-ion batteries from electric vehicles
3. Lithium-ion battery recycling
4. Leasing lithium-ion batteries in electric vehicles
5. Lithium-ion battery chemistry trends
6. Permanent magnet substitution
7. Chemical catalysts
8. Sputter targets
9. Grain boundary science of REE magnets
10. REEs in lamps (longevity)
11. Recycling of REEs in lamps
12. Hard metals cutting tools
13. Supplier diversification in electronics
14. Helium closed-loop recovery in MRI machines

Starting from this list, Consortium members were contacted to provide more information on the cases, and literature research on the cases was conducted in parallel. A table was constructed listing the following information on the cases, where available:

- CRMs of concern
- Other CRMs in the product, or that might be otherwise relevant
- Dominant circularity strategy
- Company/companies applying the strategy
- Assumed business driver (e.g. supply stability, price, sustainability)
- Challenges
- Outcomes of the strategy
- Geographic scope
- Available literature on the case

Based on the information filled in by the experts, the recommended literature was reviewed, and further literature research conducted. In further rounds of discussions within the core authoring team, 3 of the 14 case studies were selected, namely high-temperature superalloys in jet engine turbine blades, chemical processing catalysts (PGMs), and cryogenics in MRI machines (helium). The cases were selected based on two key criteria: (1) coverage of a range of industries, CRMs, and circularity strategies; and (2) sufficient information – from academic literature and/or company and government reports, and/or personal communication with relevant industry experts – to support meaningful analysis and discussion of the circularity strategies.

The case of HDD magnet recycling at Hitachi had been mentioned as a successful example in a widely cited review of REE recycling by Binnemans et al. (2013). Starting from the reference provided (Baba et al. 2013), we searched for literature and contacted the authors of the paper – who were able to provide more information. The case clearly met the criteria of diversity and available information, so it was added to the list of case studies.

It was decided that, to look beyond circularity strategies implemented in the past, at least one case should be included that reflects current challenges in the field. For this purpose, two cases seemed to be relevant and timely: recycling of traction batteries from electric vehicles, and recycling of consumer electronics. We decided on the latter because circularity strategies are documented by Apple Inc. – a company with a very popular and iconic brand in the consumer electronics industry – and the case reflects a situation lacking typical economic success factors for CRM circularity (such as high volumes of CRMs and a B2B business structure) thus posing special challenges.

The final set of five cases was taken in the core group of the authoring team, including two industry experts. After this initial selection of the cases to further observe, a case template was established for each case, comprising the preliminary findings regarding the case, its representation in the chosen framework, and open questions. These templates were sent to the Consortium, the industry focus group, and to external industry experts with knowledge from their industrial practice, for comments and corrections. The depiction of the cases in the framework was then finalized within the core authoring group.

**References**

Baba K, Hiroshige Y, Nemoto T (2013) Rare-earth magnet recycling. Hitachi Review 62:

Binnemans K, Jones PT, Blanpain B, et al (2013) Recycling of rare earths: a critical review. Journal of Cleaner Production 51:1–22. https://doi.org/10.1016/j.jclepro.2012.12.037

Blomsma F, Tennant M (2020) Circular economy: Preserving materials or products? Introducing the Resource States framework. Resources, Conservation and Recycling 156:104698. https://doi.org/10.1016/j.resconrec.2020.104698

Bocken NMP, de Pauw I, Bakker C, van der Grinten B (2016) Product design and business model strategies for a circular economy. Journal of Industrial and Production Engineering 33:308–320. https://doi.org/10.1080/21681015.2016.1172124

Ellen MacArthur Foundation (2013) Towards the circular economy: Economic and business rationale for an accelerated transition
